# c-Kit-Mediated Functional Positioning of Stem Cells to Their Niches Is Essential for Maintenance and Regeneration of Adult Hematopoiesis

**DOI:** 10.1371/journal.pone.0026918

**Published:** 2011-10-26

**Authors:** Yuki Kimura, Bisen Ding, Norikazu Imai, Daniel J. Nolan, Jason M. Butler, Shahin Rafii

**Affiliations:** 1 Department of Genetic Medicine, Weill Cornell Medical College, New York, New York, United States of America; 2 Ansary Stem Cell Institute, Weill Cornell Medical College, New York, New York, United States of America; 3 Howard Hughes Medical Institute, Weill Cornell Medical College, New York, New York, United States of America; Brigham & Women's Hospital - Harvard Medical School, United States of America

## Abstract

The mechanism by which hematopoietic stem and progenitor cells (HSPCs) through interaction with their niches maintain and reconstitute adult hematopoietic cells is unknown. To functionally and genetically track localization of HSPCs with their niches, we employed novel mutant *lox*Ps, *lox*66 and *lox*71 and Cre-recombinase technology to conditionally delete c-Kit in adult mice, while simultaneously enabling GFP expression in the c-Kit-deficient cells. Conditional deletion of c-Kit resulted in hematopoietic failure and splenic atrophy both at steady state and after marrow ablation leading to the demise of the treated adult mice. Within the marrow, the c-Kit-expressing GFP^+^ cells were positioned to Kit ligand (KL)-expressing niche cells. This c-Kit-mediated cellular adhesion was essential for long-term maintenance and expansion of HSPCs. These results lay the foundation for delivering KL within specific niches to maintain and restore hematopoiesis.

## Introduction

The molecular pathways involved in HSPC-niche cell interactions that maintain hematopoiesis during adulthood and promotes reconstitution after bone marrow (BM) suppression are not fully defined. Certain stem cell active factors are important for embryonic hematopoiesis. However, conditional deletions of these factors in adult mice do not manifest any major impairment in the HSPC maintenance, although they may serve essential functions for hematopoietic recovery after marrow suppression [1], [2], [3].

KL is expressed by BM niches raising the possibility that c-Kit/KL signaling might play a critical role in the modulation of steady state hematopoiesis [4]. In this regard, naturally-occurring *W*/c-*Kit* mutant mice have served as a great model for study of hematopoietic cell development [5]. Indeed, the encoding protein, c-Kit receptor tyrosine kinase (RTK) is employed in combination with other cell surface molecules, such as lineage^−^ (Lin^−^) and Sca-1^+^ as Lin^−^Sca1^+^c-Kit^+^ (LSK) cells, to identify enriched-population of hematopoietic stem cells [6], [7]. Nonetheless, due to the late embryonic and early neonatal lethality by the germline c-*Kit* deficiency [5], it remains to be determined whether c-Kit is essential for the maintenance and function of HSPCs in adult hematopoiesis. To address this question by genetic approach, we aimed to generate c-Kit conditional knock out (KO) mice to interrogate the role of c-Kit in hematopoietic function in adult mice. Moreover, to unravel the mechanism by which c-Kit regulates interaction of HSPCs with BM microenvironments, we develop a strategy to locate the position of the c-Kit-expressing HSPCs within the BM after conditional c-Kit deletion. To achieve these two goals, we employed mutant *lox*Ps, *lox*66 and *lox*71 [8], [9] to create a c-Kit conditional KO GFP-reporter mouse, in which deletion of c-Kit in cells results in the expression of GFP driven by the c-*Kit* native promoter. This approach allowed us to track the location of the c-Kit-deficient HSPCs within various BM niches and assess their fate at steady state conditions and during hematopoietic recovery from BM suppression.

Here, we demonstrate that c-Kit is required for hematopoiesis in the BM and spleen and the *in vivo* maintenance and regeneration of HSPCs in adult mice. Moreover, we identify that c-Kit is essential for mediating the interactions of HSPCs with niche cells *in vitro* and functional positioning of the regenerating HSPCs with KL^+^VEGFR3^+^ marrow sinusoidal endothelial cells (SECs) *in vivo*.

## Results

### Generation of c-Kit conditional KO GFP-reporter mice

**Figure 1:**
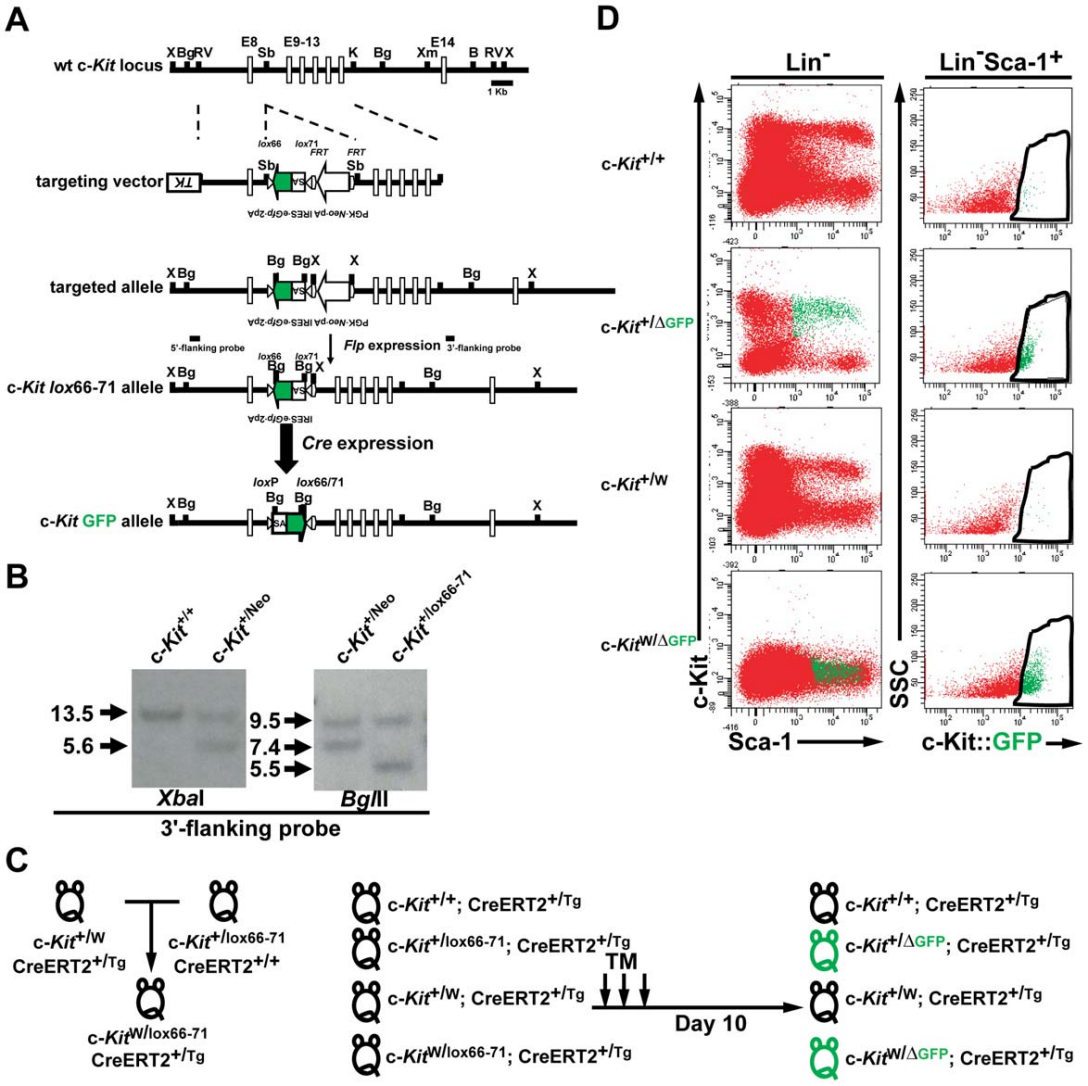
Generation of c-Kit conditional KO GFP-reporter mice by employing *lox*66–71 recombination, enabling tracking of c-Kit-deficient cells. (A) A targeting vector was designed for the insertion of a complex cassette into the 5′-region of the c-*Kit* gene. *Lox*66 and *lox*71 sites in a head-to-head orientation flank splice acceptor (SA): an inserted internal ribosomal entry site (IRES):*eGfp* as well as two polyadenylation/stop signals. Prior to Cre recombination, the c-*Kit lox*66**–**71 allele will transcribe a full-length c-*Kit* transcript. Upon activation of Cre, the inserted genes are inverted by the flanking *lox*66 and *lox*71. The IRES downstream from the c-*Kit* exon 8 allows for a bicistronic expression of *eGfp* and for an activation of stop signals from two polyadenylations, thereby creating a transcription stop of the downstream c-*Kit* gene. B, *Bam*HI; H, *Hind*III; K, *Kpn*I; RV, *Eco*RV; S, *Sal*I; Sb, *Sbf*I; *TK*, thymidine kinase; X, *Xba*I. (B) Representative Southern blot screen for ES cells and *neo*-excised ES clones. *Xba*I digestion and 3′-flanking probe were used to identify targeted ES cells. The genomic DNAs from the ES clones were digested with *Bgl*II, and the blot was probed with the 3′-flanking probe. Expected size of fragments is indicated to the left. Genotypes are indicated above each lane. (C) Schematic strategy to generate c-Kit conditional KO GFP-reporter mice. (D) Representative FACS dot plot showing the lack of c-Kit expression on Lin^−^cells from BM cells of c-*Kit* ^W/ΔGFP^ mice was confirmed by flow cytometric analysis. Left panels show the absence of c-Kit expression on Lin^−^ cells isolated from BMs of c-*Kit* ^W/ΔGFP^ mice. Green dots in the Sca-1 and c-Kit profiles gated on Lin^−^ cells represent Lin^−^Sca-1^+^c-Kit::GFP^+^ cells. Right panels show that after tamoxifen injections GFP expression is initiated in heterozygous c-*Kit* ^+/ΔGFP^ and c-Kit-deficient c-*Kit* ^W/ΔGFP^ mice. Data are representative of more than three experiments with three mice per genotype.

We generated a targeting cassette containing genes, a splice acceptor (SA) site followed by IRES-GFP-polyadenylations that were flanked by *lox*66 and *lox*71 in head-to-head orientation (Fig. 1A). The cassette was inserted in anti-sense orientation into the intron 8 of the c-*Kit* up-stream from the exon 10, which encodes the transmembrane domain of c-Kit RTK. The targeted c-*Kit lox*66-71 allele will transcribe a full-length c-*Kit* transcript, since the inserted genes remain silenced due to the anti-sense orientation relative to the c-*Kit* gene. Upon activation of Cre recombinase, the inserted genes will be flipped, but not excised, by recombination of *lox*66 and *lox*71 [8], [9], [10]. Therefore, activation of the c-*Kit* promoter leads to a splicing event with the inserted SA initiating the IRES-dependent expression of GFP, while the polyadenylation signals will silence the downstream genes, which encode the transmembrane and kinase domains of the c-Kit RTK resulting in the loss of c-Kit cell surface expression.

To generate c-Kit conditional KO GFP-reporter mice, the mutant *W* allele, which codes for a defective molecule lacking the transmembrane domain of c-Kit thereby resulting in no cell surface expression of the molecule [11], was introduced by breeding *W* heterozygotes (c-*Kit* ^+/W^ mice) to the germline-transmitted c*-Kit* ^+/lox66–71^ mice (Fig. 1B**–**C). The introduction of the mutant allele for gene targeting in the system of mutant *lox*Ps is required for circumvention of mosaic alleles which could be produced from Cre-mediated recombinations of the mutant *lox*Ps between the different alleles. The *W* heterozygotes hemizygously carried the ROSA-CreERT2 transgene (c-*Kit* ^+/W^; ROSA-CreERT2^+/Tg^ mice), allowing recombination of *lox*66 and *lox*71 sites upon tamoxifen treatment [12]. To account for any possible off-targeted phenotypes and Cre toxicity, all mice used for the following studies hemizygously carried the ROSA-CreERT2 transgene in control c-*Kit* ^+/+^, heterozygous c-*Kit* ^+/ΔGFP^, heterozygous c-*Kit* ^+/W^, and c-Kit-deficient c*-Kit* ^W/ΔGFP^ mice (Fig. 1C, Fig. S1A). All of the control CreERT2 c-*Kit* ^+/+^, CreERT2 c-*Kit* ^+/ lox66–71^, CreERT2 c-*Kit* ^+/W^, and CreERT2 c*-Kit* ^W/lox66–71^ mice were treated with equivalent doses of tamoxifen, which resulted in the generation of control CreERT2 c-*Kit* ^+/+^, CreERT2 c-*Kit* ^+/ΔGFP^, CreERT2 c-*Kit* ^+/W^ (controls), and c-Kit-deficient CreERT2 c*-Kit* ^W/ΔGFP^ mice (Fig. 1C). After day 10 of tamoxifen injection, c-Kit null phenotype on Lin^−^Sca-1^+^ cells was confirmed by flow cytometric analysis in the BM of c-*Kit* ^W/ΔGFP^ mice (Fig. 1C**–**D). Furthermore, c-Kit::GFP was expressed in both c-Kit-deficient c-*Kit* ^W/ΔGFP^ and heterozygous c-*Kit* ^+/ΔGFP^ mice, after tamoxifen treatment of c-*Kit* ^W/lox66-71^ and c-*Kit* ^+/lox66-71^ mice, respectively (Fig. 1D). The c-Kit expression level on LSK cells from the heterozygous c-*Kit* ^+/ΔGFP^ mice was reduced proportionally to that from c-*Kit* ^+/W^ heterozygoutes (Fig. 1D), while all of the pre-treated mice, c-*Kit* ^+/+^, c-*Kit* ^+/lox66–71^, c-*Kit* ^+/W^, and c-*Kit* ^W/lox66–71^ mice showed normal c-Kit cell surface expression (Fig. S1). These data strongly support the notion that the targeting strategy illustrated in Fig. 1A is efficient to delete the c-*Kit* gene and to switch on GFP expression driven by the c-*Kit* native promoter.

### Adult c-Kit deficiency alters hematopoiesis

**Figure 2:**
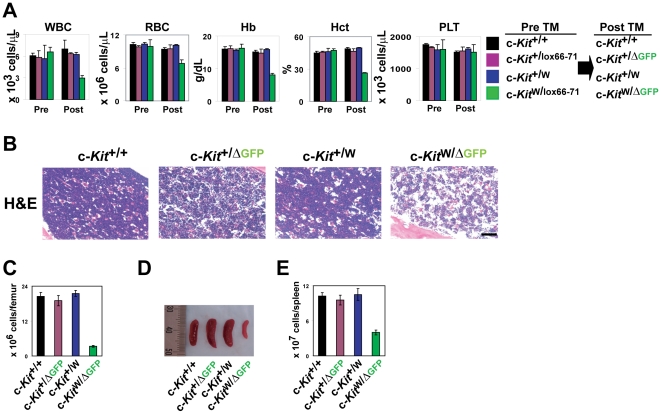
Conditional targeting of c-Kit in adult mice results in hematopoietic failures at steady state conditions. (A) c-Kit deficiency leads to impaired hematopoiesis under steady state conditions. CBC were tested in peripheral blood from each c-Kit-deficient and control mice. Data are means ± s.d. (*n* = 3). WBC, white blood cell; RBC, red blood cell: Hb, hemoglobin; Hct, hematocrit; PLT, platelet. (B) Abnormal structure of BMs. Representative images showing sections of femurs from the control and c-Kit-deficient c-*Kit* ^W/ΔGFP^ mice were stained with hematoxylin and eosin (H&E). Scale bar, 100 µm. (C) Decreased number of BMMNCs in femurs of control and c-Kit-deficient c-*Kit* ^W/ΔGFP^ mice after tamoxifen injections. Data are means ± s.d. (*n* = 3). (D) Representative image showing reduced size and progressive atrophy of spleens in c-Kit-deficient c-*Kit* ^W/ΔGFP^ mice. (E) Decreased number of splenocytes in c-Kit-deficient c-*Kit* ^W/ΔGFP^ mice. Data are means ± s.d. (*n* = 3).

The majority of mice in the series of naturally-occurring *W*/c-*Kit* mutants have macrocytic anemia [5]. Therefore, we measured complete blood counts (CBC) of the c-Kit-deficient c-*Kit* ^W/ΔGFP^ mice to investigate the effect of c-Kit deficiency on blood cell productions at steady state conditions. Although prior to tamoxifen treatment all of the CBC were normal, white and red blood cell counts as well as hemoglobin and hematocrit values in peripheral blood in the c-Kit-deficient c-*Kit* ^W/ΔGFP^ mice were significantly lower in number in comparison to those in control mice (Fig. 2A). Notably, platelet counts were normal in the c-Kit-deficient c-*Kit* ^W/ΔGFP^ mice (Fig. 2A). Furthermore, BMs of c-Kit-deficient c-*Kit* ^W/ΔGFP^ mice were hypocellular with disruption of the normal architecture (Fig. 2B-C), and there was a profound decrease in the spleen size and reduced number of splenocytes compared to those in controls (Fig. 2D**–**E). These data suggest that c-Kit is required for the maintenance of the steady state hematopoiesis and spleen mass in adult mice.

### c-Kit is required for maintenance of adult hematopoietic stem and progenitor cells

**Figure 3:**
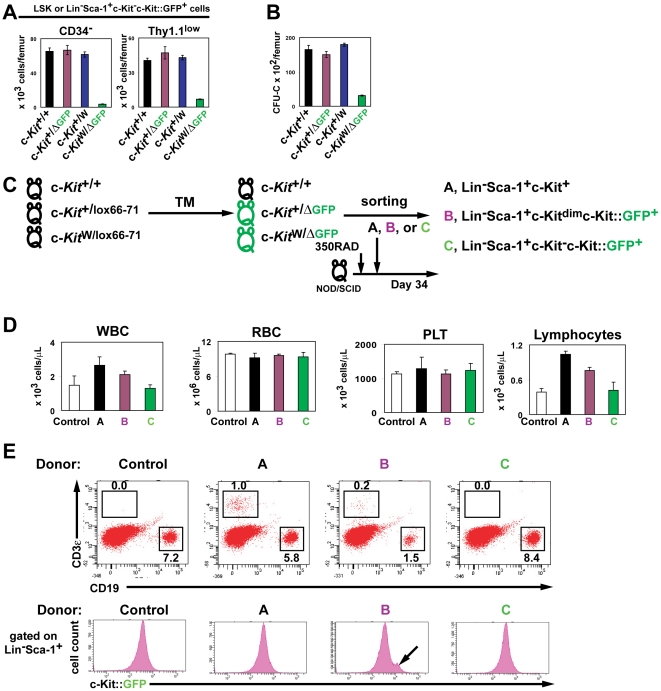
c-Kit deficiency impairs reconstitution of HSPCs in the adult mice. (A) c-Kit deficiency decreases hematopoietic stem cell subsets. Data are means ± s.d. for the numbers of CD34^−^ (left) and Thy1.1^low^ (right) cells gated on LSK or Lin^−^Sca-1^+^c-Kit^−^c-Kit::GFP^+^ cells in the BM of each genotyped mouse (*n* = 3). (B) c-Kit null affects on hematopoietic progenitor activity. Colony formation was determined with BMMNCs from control and c-Kit-deficient c-*Kit* ^W/ΔGFP^ mice. Data are means ± s.d. (*n* = 3). (C) Schematic strategy of hematopoietic reconstitution assay. Irradiated T-cell-deficient NOD/SCID mice were transplanted with LSK cells isolated from c-*Kit* ^+/+^ mice, Lin^−^Sca-1^+^c-Kit^dim^c-Kit::GFP^+^ cells isolated from heterozygous c-*Kit* ^+/ΔGFP^ mice, or Lin^−^Sca-1^+^c-Kit^−^c-Kit::GFP^+^ cells isolated from c-Kit-deficient c-*Kit* ^W/ΔGFP^ mice. (D and E) Defective hematopoietic reconstitution by c-Kit deficiency. The peripheral blood (D) and BM cells (E) from the NOD/SCID recipient mice were analyzed on day 34 after the competitive transplantation. Percentages represent the fraction of the total gated live cells within the indicated boxes. Data were obtained from or are representative of three experiments.

Next, we investigated whether the homeostasis of the phenotypically marked primitive hematopoietic cells was altered by c-Kit deficiency at the steady state conditions. Since the total cellularity of BM cells of c-Kit-deficient c-*Kit* ^WΔ/GFP^ mice was reduced in number and the c-Kit signaling is known to regulate apoptotic pathway in some cell types [13], [14], [15], we examined apoptotic status of c-Kit-deficient Lin^−^Sca-1^+^c-Kit^−^c-Kit::GFP^+^ cells. Flow cytometric analysis revealed that no apoptotic cells were detected in LSK and Lin^−^Sca-1^+^c-Kit^−^c-Kit::GFP^+^ cells from the BM of control and c-Kit-deficient c-*Kit* ^W/GFP^ mice, respectively (Fig. S2). The number of the stem cell subsets as measured by the proportions of the CD34^−^ Lin^−^Sca-1^+^c-Kit^−^c-Kit::GFP^+^ and Thy1.1^low^Lin^−^Sca-1^+^c-Kit^−^c-Kit::GFP^+^ cells in the BM of c-Kit-deficient c-*Kit* ^W/ΔGFP^ mice were significantly decreased as compared to control mice (Fig. 3A) due to the reduction of BM cellularity in the c-Kit-deficient c-*Kit* ^W/ΔGFP^ mice (Fig. 2C) although the frequencies of these cell subsets in the BM were comparable between the control and c-Kit-deficient c-*Kit* ^W/ΔGFP^ mice (data not shown). Moreover, the total number of colony-forming cells in the BM of c-Kit-deficient c-*Kit* ^W/ΔGFP^ mice was significantly decreased as compared to controls (Fig. 3B), suggesting that at steady state conditions, c-Kit is essential for the maintenance of phenotypically marked HSPCs.

To assess the hematopoietic reconstitution potential of c-Kit-deficient HSPCs, we performed competitive reconstitution assays using immunodeficient NOD/SCID mice lacking CD3^+^ T cells. LSK cells isolated from BM mononuclear cells (BMMNCs) of c-*Kit* ^+/+^ mice, Lin^−^Sca-1^+^c-Kit^dim^c-Kit::GFP^+^ purified from BMMNCs of heterozygous c-*Kit* ^+/ΔGFP^ mice or c-Kit-deficient Lin^−^Sca-1^+^c-Kit^−^c-Kit::GFP^+^ cells isolated from BMMNCs of c-Kit-deficient c-*Kit* ^W/ΔGFP^ mice, were transplanted with BMMNCs from naïve NOD/SCID mice into irradiated-recipient NOD/SCID mice (Fig. 3C). Quantification CBC in the recipient mice on day 34 after transplantation revealed that the numbers of white blood cells and lymphocytes were increased in peripheral blood of the recipient mice transplanted with either LSK or Lin^−^Sca-1^+^c-Kit^dim^c-Kit::GFP^+^ cells (Fig. 3D). This suggests that the transplanted HSPCs engrafted in the BM of the recipient mice and gave rise to lymphocyte cell subsets, which lacked in NOD/SCID mice. However, those numbers in the recipient mice transplanted with c-Kit-deficient Lin^−^Sca-1^+^c-Kit^−^c-Kit::GFP^+^ cells were not increased compared to controls (Fig. 3D). Additionally, the flow cytometric analyses revealed that CD3^+^ T cells were reconstituted in the recipient NOD/SCID mice transplanted with LSK or Lin^−^Sca-1^+^c-Kit^dim^c-Kit::GFP^+^ cells, while no CD3^+^ T cells were detected in the recipient NOD/SCID mice transplanted with c-Kit-deficient Lin^−^Sca-1^+^c-Kit^−^c-Kit::GFP^+^ cells (Fig. 3E). Notably, c-Kit::GFP^+^ cells were reconstituted in the BM of the recipient NOD/SCID mice transplanted with Lin^−^Sca-1^+^c-Kit^dim^c-Kit::GFP^+^ HSPCs, but not in NOD/SCID recipients transplanted with c-Kit-deficient Lin^−^Sca-1^+^c-Kit^−^c-Kit::GFP^+^ HSPCs (Fig. 3E). These results indicate that c-Kit-deficient LSK cells fail to reconstitute hematopoiesis in the irradiated recipient mice, suggesting that c-Kit activation is essential for hematopoietic reconstitution.

### c-Kit mediates interaction of HSPCs with niche cells

**Figure 4:**
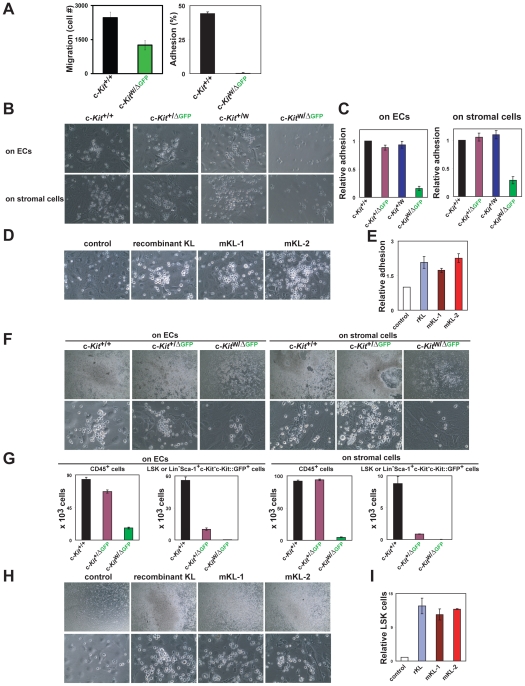
c-Kit regulates interactions of HSPCs with endothelial and BM stromal cells. (A) Dramatic reduction of migration activity and adhesion to microenvironment by c-Kit deficiency. Lin^−^ BM cells from c-*Kit* ^+/+^ and c-Kit-deficient c-*Kit* ^W/ΔGFP^ mice were used for migration assay and assessment of the extent of adhesion to laminin. Data are means ± s.d. (*n* = 3). (B) c-Kit-deficient Lin^−^ BMMNCs fail to form colonies on ECs and stromal cells. Representative images were obtained from three independent experiments. Magnification, x 40. (C) c-Kit-deficient Lin^−^ BMMNCs failed to adhere onto ECs and stromal cells. Adherent LSK or Lin^−^Sca-1^+^c-Kit^−^c-Kit::GFP^+^ HSPCs were quantified by flow cytometry. The ratios of adhered LSK cells from c-*Kit* ^+/+^ mice were considered as 1. Data are means ± s.d. (*n* = 3). (D) KL supports the adhesion and the colony formation of Lin^−^ BMMNCs onto ECs. Lin^−^ BMMNCs from wild-type mice were transferred onto ECs, which overexpressed mKL-1 or mKL-2. Representative images were obtained from three independent experiments. Magnification, x 40. (E) KL-c-Kit signaling enhances the adhesion of Lin^−^ BMMNCs onto the ECs. Adherent LSK cells were analyzed by flow cytometry. The ratios of adherent LSK cells onto control ECs were considered as 1. Data are means ± s.d. (*n* = 3). (F) c-Kit-deficient Lin^−^ BMMNCs fail to produce immature HSPCs on ECs and stromal cells. The co-cultivations were performed as described above. Representative images were obtained from three experiments. Upper panels, x 4 magnification. Lower panels, x 40 magnification. (G) c-Kit deficiency fails to produce immature HSPCs. CD45^+^, LSK, and c-Kit-deficient Lin^−^Sca-1^+^c-Kit^−^c-Kit::GFP^+^ cells were quantified by flow cytometry after 10 days of coculture. Data are means ± s.d. (*n* = 3). (H) KL supports expansion of LSK cells. Lin^−^ BMMNCs from wild-type mice were transferred onto ECs, which overexpressed either mKL-1 or mKL-2. Representative images were obtained from three experiments. Upper panels, x 4 magnification. Lower panels, x 40 magnification. (I) KL-c-Kit signaling enhances expansion of LSK cells cocultured with ECs. Adherent LSK cells were analyzed by flow cytometry. The ratios of adherent LSK cells onto control ECs were considered as 1. Data are means ± s.d. (*n* = 3).

Cellular adhesion of HSPCs with prototypical BM niche cells, such as endothelial and stromal cells could play a major role in maintenance of hematopoiesis. Therefore, we employed *in vitro* adhesion and migration assays to determine whether c-Kit activation is essential for proper interaction of the HSPCs with the niche cells. To this end, lineage-depleted hematopoietic cells (Lin^−^ cells) isolated from c-Kit-deficient c-*Kit* ^W/ΔGFP^ and control mice were used for Boyden chamber assay and adhesion assay to laminin, an extracellular matrix component that is deposited by niche cells. Lin^−^ cells derived from c-Kit-deficient c-*Kit* ^W/ΔGFP^ mice manifested severe defects in migration and adhesion as compared to Lin^−^ cells derived from c-*Kit* ^+/+^ mice (Fig. 4A). These data indicate that c-Kit is required for migration and adhesion of HSPCs to niche cells.

To define the mechanism by which c-Kit deficiency results in the impairment of hematopoiesis, we interrogated the role of c-Kit in mediating interaction of HSPCs with BM niche cells [4], [16], [17], [18], [19], [20], [21]. Specifically, we hypothesized that c-Kit might regulate adhesion and/or migration of HSPCs to BM endothelial and stromal cells. To test this hypothesis, we determined the effect of c-Kit deficiency on adhesion and migration activities of HSPCs using c-Kit-deficient cells purified from BMs of c-*Kit* ^W/ΔGFP^ mice, in a coculture system with niche cells [22]. Exogenous sKL stimulated adhesion of Lin^−^ cells derived from the control mice onto both primary endothelial cells (ECs) and BM-derived stromal cells, cocultured in serum- and cytokine-free condition. The cocultured hematopoietic cells formed typical cobblestone colonies on endothelial and stromal cells, while c-Kit-deficient Lin^−^ cells from BMs of c-*Kit* ^W/ΔGFP^ mice detached from endothelial and stromal cells and failed to form adherent or migrating colonies (Fig. 4B). In addition, some cells were observed to tightly adhere to ECs and transmigrated underneath the ECs. The ratio of c-Kit-deficient Lin^−^Sca-1^+^c-Kit^−^c-Kit::GFP^+^ HSPCs that interact with niche cells were much lower than those of wild-type LSK HSPCs (Fig. 4C). These data suggest that c-Kit signaling stimulates tight interactions of HSPCs with niche cells [21], [23].

Integrin molecules expressed on cell surface are known to provide tight adhesion between cells. Unexpectedly, there were no significant difference in cell surface expressions of α4, α5, α6, and β1 integrins on between LSK and c-Kit-deficient Lin^−^Sca1^+^c-Kit^−^c-Kit::GFP^+^ cells (Fig. S3). However, as membrane KL (mKL) supports interaction of mast cells with stromal cells [11], [24], we speculated that mKL expressed on niche cells might also support interactions of c-Kit-expressing cells to the various spliced isoforms of KLs expressed on niche cells. After undergoing splicing KLs generate the membrane isoforms, mKL-1 and mKL-2, which lacks the metalloproteinase-cleavage site (Fig. S4) [25], [26]. Each KL isoform supported the tight adhesion of wild-type Lin^−^ BMMNCs onto ECs (Fig. 4D**–**E), suggesting that the HSPC-niche cell interaction is enhanced by the KL-c-Kit signaling. To investigate whether the c-Kit-mediated interaction was important for maintenance and expansion of HSPCs, we extended the co-cultivation period. There was an incremental expansion of LSK cells purified from BMs of c-*Kit* ^+/+^ mice over a 10 day culture period (Fig. 4F**–**G). By contrast, c-Kit-deficient Lin^−^Sca-1^+^c-Kit^−^c-Kit::GFP^+^ cells failed to form colonies or proliferate in co-culture with endothelial and stromal cells (Fig. 4F**–**G). In addition, mKL-1 or mKL-2 expressed on ECs supported expansion of large numbers of LSK cells (Fig. 4H**–**I). These findings indicate that the KL-c-Kit interaction accelerates motility and adhesion of HSPCs to the ECs and stromal cells thereby supporting their long-term expansion.

### c-Kit is essential in regeneration of adult hematopoiesis

**Figure 5:**
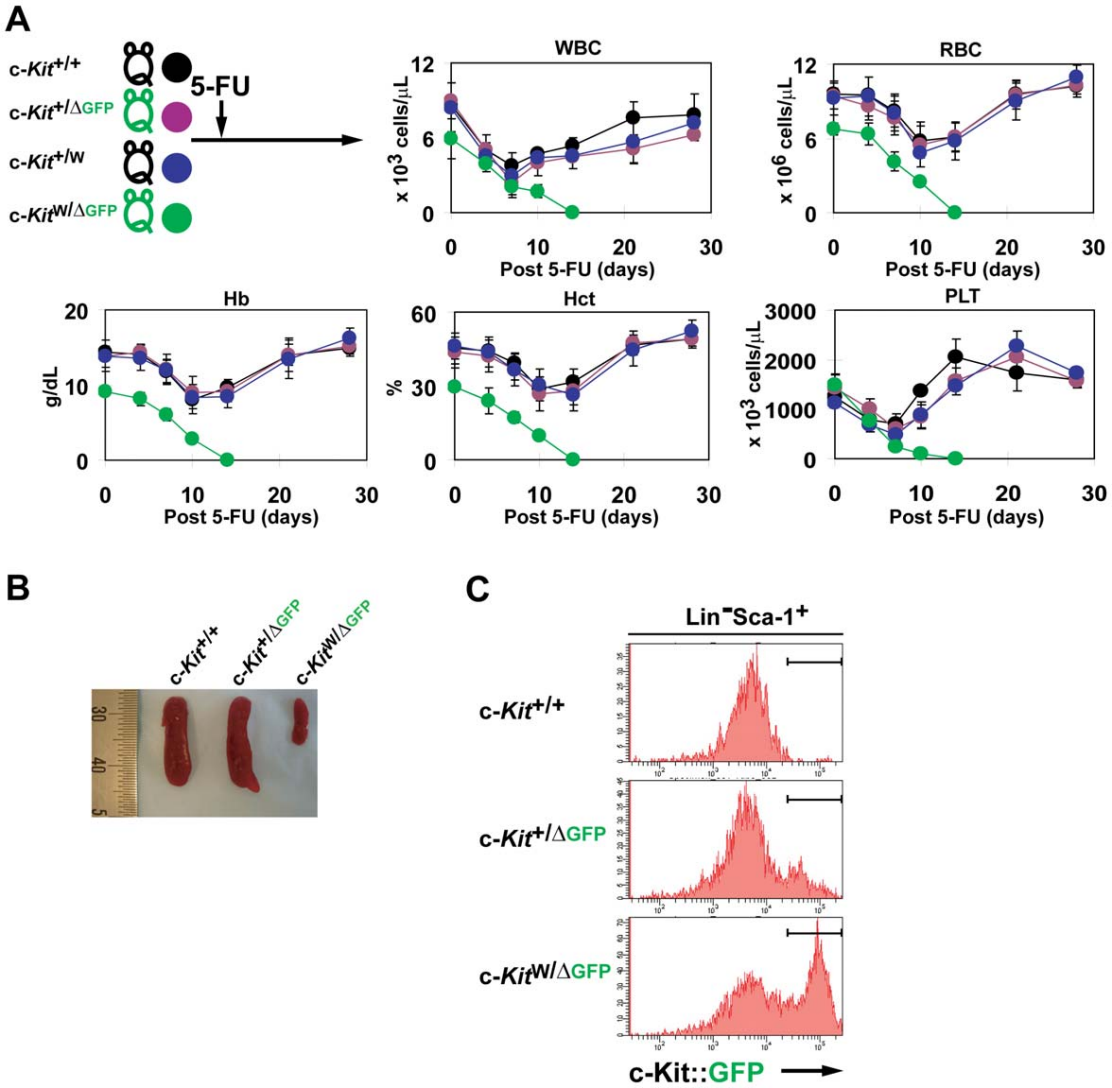
c-Kit-deficient mice fail to reconstitute hematopoietic cells after 5-FU induced marrow depletion. (A) CBC from 5-FU treated mice were monitored on each time point during hematopoietic recovery from BM depletion. Data are means ± s.d. (*n* = 3). (B) Lack of c-Kit results in severe impairment in increase of the spleen size. (C) c-Kit-deficient HSPC-rich population is present in the BM of c-*Kit* ^W/ΔGFP^ mice on day 14 after 5-FU injection. Data were obtained from or are representative of two independent experiments with three mice per genotype.

To determine whether c-Kit-mediated interaction of HSPCs with the BM microenvironment is essential for hematopoietic recovery and conferring HSPCs resistance to BM suppression [16] in adult mice, we assessed the capacity of the c-Kit conditional KO GFP-reporter mice to recover from high dose 5-FU mediated BM suppression [27]. Although there was a complete recovery of CBC in the control mice, in the c-Kit-deficient c-*Kit* ^W/ΔGFP^ mice there was a severe impairment in the hematopoietic recovery resulting in the death of the mice due to persistent pancytopenia by day 14 after 5-FU treatment (Fig. 5A). There was no major obvious organ toxicity observed in the vital organs of the 5-FU-treated mice (data not shown). Under BM suppressive conditions, c-Kit-deficient c-*Kit* ^W/ΔGFP^ mice did not reveal the rebound extramedullary splenomegaly, which was observed in control mice (Fig. 5B). The spleen size was significantly reduced and atrophied in c-Kit-deficient c-*Kit* ^W/ΔGFP^ mice as compared to even the control untreated mice. Furthermore, on day 14 after BM suppression, c-Kit-deficient Lin^−^Sca-1^+^c-Kit^−^c-Kit::GFP^+^ HSPCs were decreased in number (data not shown). Notably, in the BM of c-Kit-deficient c-*Kit* ^W/ΔGFP^ mice the GFP expression was enhanced compared to that in heterozygous c-*Kit* ^+/ΔGFP^ mice on day 14 after the 5-FU injection (Fig. 5C). These data suggest that c-Kit activation is essential for hematopoietic reconstitution after BM myeloablation.

### c-Kit regulates interactions of HSPCs to KL^+^VEGFR3^+^ sinusoidal ECs (SECs) in the BM

**Figure 6:**
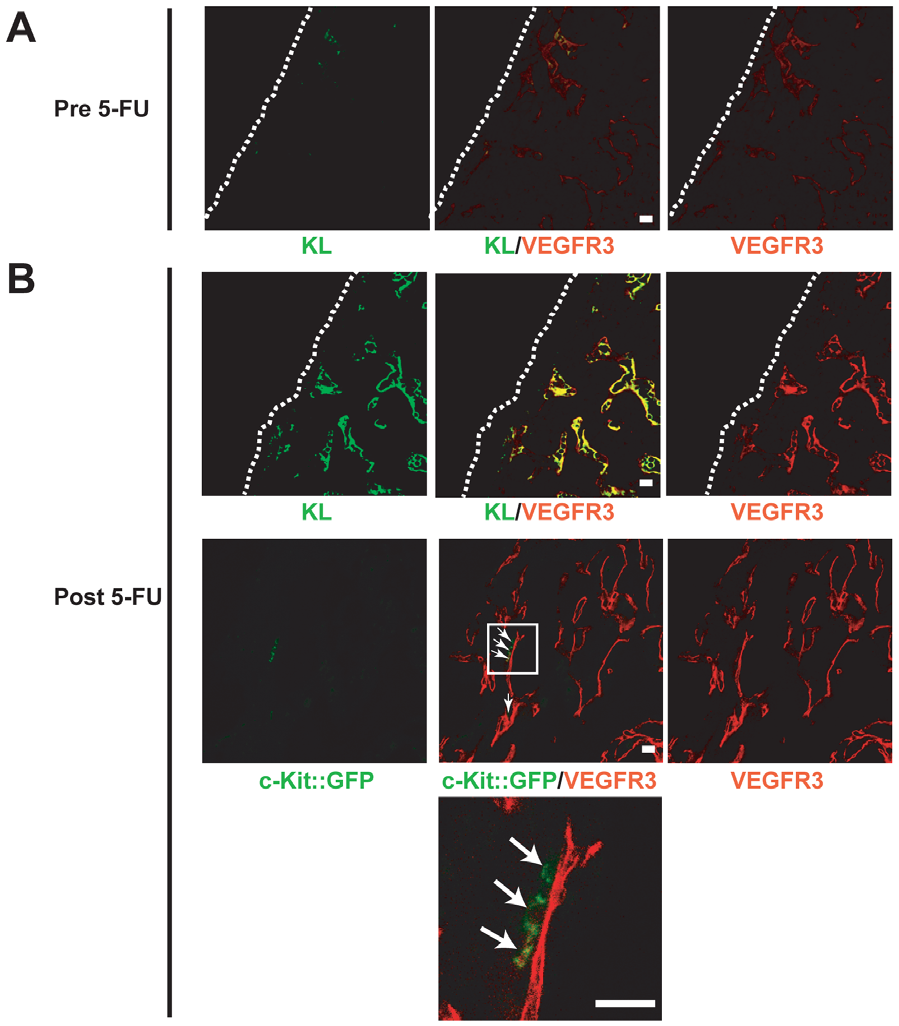
c-Kit::GFP^+^ cells are positioned in close vicinity to KL^+^VEGFR3^+^ sinusoidal ECs. Femurs from c-Kit-deficient c-*Kit* ^W/ΔGFP^ mice on day 0 (A) and 14 (B) during BM depletion were co-stained for VEGFR3 (red), which demarcates SECs. Arrows indicate c-Kit-deficient c-Kit::GFP^+^ cells. The dotted lines represent the border between the bone and hematopoietic tissue. The lower image is magnified from the enclosed box. Scale bars, 20 µm.

The advantage of the *lox*66*-lox*71 Cre-recombinase conditional KO GFP-reporter strategy allows us to precisely localize the c-Kit-deficient c-Kit::GFP^+^ cells in the BM during the recovery from BM suppression. Indeed, c-Kit-deficient c-Kit::GFP^+^ cells were detected in BMs of the 5-FU-treated c-Kit-deficient c-*Kit* ^W/ΔGFP^ mice (Fig. 6). Using VEGFR3 as a marker for SECs in the BM [27], confocal analysis demonstrated that c-Kit-deficient c-Kit::GFP^+^ cells were close cellular apposition of but detached from VEGFR3^+^ SECs (Fig. 6B). Although at steady state conditions the expression of mKL was low on VEGFR3^+^ SECs (Fig. 6A), after BM suppression there was a significant upregulation of mKL expression on VEGFR3^+^ SECs in the BM (Fig. 6B). These data suggest that interaction of HSPCs with the SECs enhanced by KL-c-Kit signaling could be important for hematopoietic recovery.

## Discussion

We have employed a *lox*66*-lox*71 Cre-recombinase conditional KO approach to dissect c-Kit functions in adult mice. This strategy not only results in c-Kit deletion but also by genetic labeling of the c-Kit-deficient cells with GFP, facilitates isolation of these cells for *in vitro* studies and imaging of the c-Kit-deficient cells *in vivo*. We demonstrate for the first time that c-Kit expression is required for adult hematopoiesis within the BM and spleen at the steady state conditions and hematopoietic recovery from BM depletion. Furthermore, we show that c-Kit signaling enhances the interaction of HSPCs with niche cells, allowing for proper functional positioning of the repopulating HSPCs within the BM microenvironment.

Among the known RTKs, expression of Tie2, VEGFR2, VEGFR1, and FGF-receptors have been implicated in modulating the homeostasis of HSPCs during the development. However, none of these RTKs have been shown to play a major role in mediating the homeostasis of HSPCs in adult mice at steady state conditions. Therefore, c-Kit is one of the critical RTKs that modulates the steady state and long-term maintenance of HSPCs. Here, we demonstrate that both at steady state and after BM depletion, KL enhances the interaction of c-Kit-expressing cells with KL^+^ niche cells. The mKL-c-Kit pair not only performs as a survival pathway but also allows for functional positioning of HSPCs to the various BM niches to maintain HSPCs and accelerate hematopoietic recovery. Notably, both at steady state conditions and after 5-FU mediated BM depletion in the c-Kit-deficient mice there is a profound decrease in the size of spleen, with a major defect in physiological increase in the spleen size after BM depletion. It is conceivable that during hematopoietic recovery the atrophy in the size of spleen might be due to impaired mobilization of HSPCs from the BM to the spleen. Alternatively, c-Kit activation may be essential for expansion and maintenance of HSPCs within the spleen. Mutations of the juxtamembrane domain tyrosines in c-Kit RTK results in splenic hypertrophy but in normal hematocrit values in adult mice due to the loss-of-docking sites for their particular target molecules and reduced kinase activity [28]. Thus, at steady state conditions, proper c-Kit signaling is essential to maintain the spleen mass supporting the homeostasis of HSPCs.

We have taken advantage of *lox*66-*lox*71 conditional knock down of the c-Kit in adult mice, enabling simultaneous tracking of HSPCs, which lack c-Kit in the BM during the steady state and after 5-FU hematopoietic suppression. The finding that c-Kit expression regulates hematopoiesis and spleen size in adult mice even at the steady state has major clinical implications, setting forth the concept that deregulations of KL-c-Kit signaling results in hematopoietic failure states in a wide variety of clinical disorders.

## Materials and Methods

### Targeting construct

The fragment containing pGK-HSV-TK was excised from pDELBOY (a gift from D. J. Rossi, Stanford) and inserted between *EcoR*I and *Not*I sites of pBluescript II SK (+) (Stratagene, La Jolla, CA). The plasmid was named A-1_TK-IN. Then a 6.1 KB *EcoR*V- *Kpn*I fragment of C57BL/6 genomic DNA containing exons 8**–**13 of the c-*Kit* gene from a bacterial artificial chromosome clone (RP23-142L11) was ligated with a *Cla*I linker and subsequently inserted at the sites of *Cla*I and *Kpn*I of the A-1_TK_IN. The product was named A-2-c-kit-IN. Two synthesized oligos (5′-CCTGCAGGATAACTTCGTATAGCATACATTATACGAACGGTATCCCGGGGGGATGTTCGGGCGGCCGCCCATAACGGAAGATCTTCCATAACTTCGTATAATGTATGCTATACGAACGGTATTAATTAACGATGCCCACATGAGCTCCCTGCAGGTAGCT-′3; 3′-CATGGGACGTCCTATTGAAGCATATCGTATGTAATATGCTTGCCATxAxGGGCCCCCCTACAAGCCCGCCGGCGGGTATTGCCTTCTAGAAGGTATTGAAGCATATTACATACGATATGCTTGCCATAATTAATTGCTACGGGTGTACTCGAGGGACGTCCA-′5) containing *Sbf*I, *lox*66, *Xma*I, *Not*I, *Bgl*II, *lox*71, *Pac*I, *Sac*I, and *Sbf*I sites were annealed and subsequently introduced at the sites of *Kpn*I and *Sac*I of pBluescript II SK (+). The plasmid was named B-1_Adapter-IN. A fragment containing an internal ribosomal entry site (IRES) and eGFP excised from pccall2-IRES-EGFP (a gift from A. Nagy, Samuel Lunenfeld Research Institute) was subcloned into the B-1_Adaptor-IN at the *Bgl*II and *Not*I sites. The product was named B-2_IRES-EGFP-IN. A fragment containing splice acceptor (SA) from pBS-SA (A1) (a gift from T. Tanaka, UIUC) was introduced into the sites of *Bgl*II and *Cla*I of the B-2_IRES-EGFP-IN. The product was named B-2-1_SA-IN. A fragment containing pGK pA and pGH pA from 2xpA-1 in pGEM-T (a gift from A. Nagy) was introduced into the sites of *Not*I and *Xma*I of the B-2-1_SA-IN. The product was named B-3-1_2xpA-IN. A *Sac*I-*Pvu*I fragment containing a *neomycin* resistance gene (*Neo*) flanked by *Frt* from the pDELBOY was subcloned at the *Sac*I and *Pac*I sites of the B-3-1_2xpA-IN. The product was named B-4-1_Frt-PGK-Neo-IN. The *Sbf*I-*Sbf*I fragment containing Frt-flanking the *Neo* and *lox*66-71-flanking the SA, IRES, EGFP, and two pA genes, was excised from the B-4-1_Frt-PGK-Neo-IN and was inserted at the *Sbf*I site of the A-2_c-kit-IN. The final product is a targeting construct, named A-3-1-B4-IN.

### Mice

The targeting vector was linearized by *Ahd*I and then transfected by electroporation into C2J ES cells derived from C57BL/6-*Tyr* ^c-2J^/J mice. Colonies doubly resistant for G418 and gancyclovir were screened by Southern blot analysis for homologous recombination with *Xba*I digestion using 3′-flanking probe. Subsequently, the *Neo* was excised from the targeted allele in the homologous recombinant ES clones by transient expression of Flippase-puromycin plasmid. After screening of *Neo*-excised ES clones by Southern blot hybridization with *Bgl*II digestion using 3′-flanking probe, the screened ES clones were used for microinjections with C57BL/6 blastocysts. Generated chimeric mice were bred with C57BL/6-*Tyr* ^c-2J^/J female mice, and their albino-looking offspring were genotyped to confirm germline transmission of the c-*Kit lox*66**–**71 allele. The ROSA-CreERT2 mice (CD1 back ground) were obtained from T. N. Sato at Weill Cornell Medical College (WCMC), NY. C57BL/6-*Tyr* ^c-2J^/J, NOD/SCID, and C57BL/6-background c-*Kit* ^+/W^ mice were purchased from Jackson Laboratory (Bar Harbor, ME).

### c-Kit conditional KO GFP-reporter mice

For generation of c-*Kit* ^W/lox66-71^ mice, the c-*Kit* ^+/lox66-71^ mice were crossed with heterozygous c-*Kit* ^+/W^ mice carrying hemizygous ROSA CreERT2 transgene. The mice hemizygously carrying the ROSA CreERT2 transgene were treated with tamoxifen at a dose of 450 mg/Kg body i.p. for three days. The treated mice on day 10**–**14 post tamoxifen were used for the experiments. All mice were maintained in specific pathogen-free conditions in the Animal Facility of WCMC. All experiments were approved by the Institutional Animal Care and Use of Committee of WCMC (protocol #: 2009-0061).

### Genotyping

For the detection of the c-*Kit lox*66**–**71 allele, mice were genotyped using Primer a and Primer b (Primer a, 5′-CCC GGA GCC CAC AAT AGA TTG-′3; Primer b, 5′-AAC CAG CTG GGG CTC GAA ATT-′3). For genotyping of the c-*Kit* GFP allele, mice were genotyped using Primer a and Primer c (Primer c, 5′-CGG GCC CTC ACA TTG CCA AAA-′3). For genotyping of the c-*Kit* wild-type allele, mice were genotyped using Primer a and Primer d (Primer d, 5′-CTG TCC TGG GAA ATT GCT TTA-′3). PCR reactions contained 1 x PCR buffer containing 1.5 mM MgCl_2_ (Denville Scientific, South Plainfield, NJ), 200 µM deoxynucleotides (Invitrogen, Carlsbad, CA), 0.5 µM primers, and 1 U of Taq DNA polymerase (Denville Scientific). Cycling conditions were as follows: 94°C for 5 minutes, 35 cycles of 94°C for 1 minute/62°C for 1 minute/72°C for 2 minutes. Presence of the c-*Kit lox*66**–**71 and GFP alleles resulted in a 406 bp band and a 644 bp band, respectively. The c-*Kit* wild-type allele was detected by a 362 bp band. For detection of the *cre* transgene, mice were genotyped using primers, Cre S01 and Cre A01 (Cre S01, 5′-CCAAAATTTGCCTGCATTACCGGTCGATGC-′3; Cre A01, 5′-AGCGCCGTAAATCAATCGATGAGTTGCTTC-′3). Reaction conditions were as described above. Cycling conditions were 94°C for 5 minutes, 35 cycles of 94°C for 30 seconds/58°C for 30 seconds/72°C for 2 minutes, followed by 72°C for 10 minutes.

### Isolation of cells and flow cytometry

BMMNCs were obtained from either flushing or crushing femurs, tibiae, and humerus. They were suspended in MOPS buffer (Miltenyi Biotech, Inc., Bergisch Gladbach, Germany) and filtered through nylon screen (45 µm, Sefer America, Kansas City, MO) to obtain a single cell suspension. Cells were counted using a hemocytometer. Alive non-stained cells with the DNA-intercalating dye 4,6-diamidine-2-phenylindole (DAPI, Sigma, St. Louis, MO) were analyzed by LSRII-SORP equipped with 355-nm, 405-nm, 488-nm, and 633-nm lasers and FACSDiva 6.1 software (BD Bioscience, San Jose, CA). All antibodies were purchased from either BD Bioscience or eBioscience (San Diego, CA). BD; biotin mouse lineage panel (CD3e (145-2C11), CD11b (M1/70), B220 (RA3-6B2), Gr-1 (RB6-8C5), and TER-119 (TER-119)), c-Kit (2B8), Sca-1 (D7), CD45.2 (104), rat IgG2a (R35-95), rat IgG2b (A95-1), Thy1.1 (OX-7), CD34 (RAM34), α4 (R1-2), CD16/CD32 (2.4G2), CD3ε(145-2C11), CD19 (1D3), CD11b (M1/70), and AnnexinV. eBioscience; α5 (HMa5-1), α6 (GoH3), β1 (HMb1-1), and Armenian hamster IgG (eBio299Arm). Lineage-depleted cells were prepared using Lineage Cell Depletion Kit Mouse (Miltenyi Biotech). For purification of cell subsets, the lineage-depleted cells were used for sorting with FACS Aria flow sorter (BD).

### CBC

Retro-orbital peripheral blood was collected into microhematocrit capillary tubes (Fisher Scientific, Pittsburgh, PA). CBC were counted by Bayer Advia 120 Multi-species Hematology Analyzer with multispecies software (Bayer HealthCare, Tarrytown, NY).

### Competitive reconstitution

Sub-lethally irradiated (350 Rads) NOD/SCID mice were competitively reconstituted with 3×10^3^ purified LSK, Lin^−^Sca-1^+^c-Kit^dim^c-Kit::GFP^+^, or Lin^−^Sca-1^+^c-Kit^−^c-Kit::GFP^+^ cells from lineage-depleted BMMNCs from c-*Kit* ^+/+^, heterozygous c-*Kit* ^+/ΔGFP^, or c-Kit-deficient c-*Kit* ^W/ΔGFP^ mice, respectively, in competition with 2×10^5^ BMMNCs from naïve NOD/SCID mice. CBC and BMMNCs from recipient mice were analyzed on day 34 after the transplantation. 

### Methylcellulose culture

The colony forming was assayed using MethoCult M3434 (Stem Cell Technologies, Vancouver, Canada).

### Organ histology and immunofluorescence

All organs were fixed in 4% paraformaldehyde (PFA). Femurs were decalcified in 10% EDTA in PBS containing Ca and Mg and then transferred into either 30% ethanol for preparation of paraffin sections or 30% sucrose for preparation of frozen sections. The treated tissues were embedded in OCT medium and frozen in liquid nitrogen. Hematoxylin and eosin (H&E) stains were done by Histoserv (Germantown, MD). For IF, the frozen sections (10 µm) were blocked with 10% normal donkey serum/0.05% Tween 20 in PBS containing Ca and Mg for 2 hr at RT followed by primary antibody reactions for over night at 4°C. On the next day, the stained sections were reacted with proper secondary antibodies for 1 hr at RT and then stained with DAPI (Invitrogen) for 20 min at RT for nuclei counter staining. Fluorescent images were obtained using Carl Zeiss LSM710 system (Carl Zeiss, Thornwood, NY). The primary and fluorophore-conjugated secondary antibodies (Abs) were anti-KL (Santa Cruz, Santa Cruz, CA), anti-VEGFR3 (mF4-31c1, a gift from ImClone Systems, New York, NY), anti-GFP conjugated with biotin (Invitrogen), and fluorophore-conjugated Abs (Jackson ImmunoResearch Laboratories, West Grove, PA).

### Migration assay

1×10^5^ lineage-depleted BMMNCs in 100 µL 0.1% BSA IMDM (Invitrogen) were placed in the top compartments and 600 µL medium containing 50 ng/mL mouse recombinant SDF-1α (Pepro Tech Inc., Rocky Hill, NJ) in the bottom chambers of the 5.0 µm transwell plates (Corning, Corning, NY). After 6 hr at 37°C in a 5% CO_2_ incubator, the migrated cells in the bottom chambers were harvested and then measured in number using Cell Titer-Glo Luminescent Cell Viability Assay (Promega, Madison, WI).

### Cells and lentivirus infection

ECs and BM-derived stromal cells were prepared as described [29]. ECs were infected with lentivirus. Lentivirus vector (pCCL-pGK-GFP-WPRE, a gift from S. Rivella, WCMC) was subcloned with cDNA of mKL-1 or mKL-2 which were cloned from wild-type C57BL/6 splenocytes using primers as described [25]. The RNAs were extracted the infected ECs using RNeasy Mini Kit (Qiagen Science, Valencia, CA), and cDNAs were synthesized with SuperScript reverse transcriptase (Invitrogen). qPCR (Taqman) was done as described [30]. Primers, mKitL-F, 5′-TGCGGGAATCCTGTGACTGATAATG-′3; mKitL-R, 5′-CAAAACATCCATCCCGGCGAC-′3.

### Adhesion and expansion assays

For adhesion and expansion assays of HSPCs to ECs and BM stromal cells, 2.5×10^5^ Lin^−^ BMMNCs were placed onto either ECs or BM-derived stromal cells in non-serum X-VIVO 20 medium (Lonza, Walkersville, MD) with or without 20 ng/mL mouse recombinant KL (Pepro Tech Inc.) in 12-well plates. After 18 hr or 10 day culture, adhered cells were harvested by mild treatment of trypsin and then counted in cell numbers. The number of LSK or c-Kit-deficient Lin^−^Sca-1^+^c-Kit^−^c-Kit::GFP^+^ cells were calculated by frequencies of these cells quantified with a flow cytometry LSRII and the counts of BMMNCs with hemocytometer. In-put LSK or c-Kit-deficient Lin^−^Sca-1^+^c-Kit^−^c-Kit::GFP^+^ cells were also calculated by the same way, and the % adhesion was determined by the number of the adhered cells/the number of the in-put cells. For adhesion assay to laminin, 1×10^5^ Lin^−^ BMMNCs in 100 µL 0.1% BSA IMDM containing 10 ng/mL mouse recombinant KL were placed in laminin-coated wells of 96-well plates (BD Bioscience). After 1 hr incubation at 37°C in a 5% CO_2_ incubator, non-adherent cells were washed out two times by PBS. The adherent cells were quantified in number using Cell Titer-Glo Luminescent Cell Viability Assay. The % adhesion was calculated by the number of adherent cells/in-put cell numbers.

### BM suppressive treatment

Mice were injected with 5-FU (250 mg/Kg body weight, Sicor Pharmaceutical Inc., Irvine, CA) intravenously [27].

## Supporting Information

Figure S1 **Normal LSK cells in c-Kit conditional KO GFP-reporter mice in pre-activation of Cre recombinase.** (A) Normal LSK cells are present in the BM of c-*Kit* ^W/lox66-71^ mice at the time of pre-treatment with tamoxifen. The schema shows breeding strategy to generate c-*Kit* ^W/lox66–71^ mice carrying ROSA-CreERT2 transgene as well as control ROSA-CreERT2 c-*Kit* ^+/+^, ROSA-CreERT2 c-*Kit* ^+/lox66–71^, and ROSA-CreERT2 c-*Kit* ^+/W^ mice. The flow cytometric analysis demonstrated that normal population of LSK cells manifests no GFP expression in the BM of ROSA-CreERT2 c-*Kit* ^+/+^, ROSA-CreERT2 c-*Kit* ^+/lox66–71^, ROSA-CreERT2 c-*Kit* ^+/W^, and ROSA-CreERT2 c-*Kit* ^W/lox66–71^ mice. The flow cytometric data are representative of each genotype. (B) Normal number of BMMNCs in femurs of ROSA-CreERT2 c-*Kit* ^+/+^, ROSA-CreERT2 c-*Kit* ^+/lox66–71^, ROSA-CreERT2 c-*Kit* ^+/W^, and ROSA-CreERT2 c-*Kit* ^W/lox66–71^ mice at the time of pre-treatment with tamoxifen. Data are means ± s.d. (*n* = 3).
(TIF)

Figure S2 **No apoptosis was detected in c-Kit-deficient Lin^−^Sca-1^+^c-Kit^−^c-Kit::GFP^+^ cells in the BM of c-** ***Kit*** **^W/ΔGFP^ mice.** Apoptotic status was examined in LSK or Lin^−^Sca-1^+^c-Kit^−^c-Kit::GFP^+^ cells in the BM of c-*Kit* ^+/+^, heterozygous c-*Kit* ^+/ΔGFP^, heterozygous c-*Kit* ^+/W^ mice, and c-Kit-deficient c-*Kit* ^W/ΔGFP^ mice by staining with AnnexinV. As a positive control, wild-type mice were irradiated with 950 Rads, and BM cells from the mice on day 3 post radiation were analyzed for apoptotic status. Results are mean percentages ± s.d. of AnnexinV^+^ cells gated on DAPI**^−^** cells derived from three mice.
(TIF)

Figure S3 **Normal expressions of integrins on HSPCs lacking c-Kit expression.** The flow cytometric data show expressions of α4, α5, α6, and β1 integrins on LSK or Lin^−^Sca-1^+^c-Kit^−^c-Kit::GFP^+^ cells in the BM of c-*Kit* ^+/+^, heterozygous c-*Kit* ^+/ΔGFP^, heterozygous c-*Kit* ^+/W^, and c-Kit-deficient c-*Kit* ^W/ΔGFP^ mice. Data are representative of more than three experiments with three mouse per genotype.
(TIF)

Figure S4 **Overexpression of KLs on ECs.** (A) Illustrations show diagrammatic structures of series of KLs and a backbone of the lentivirus vectors. An asterisk indicates the position of the proteolytic cleavage site. mKL-1, membrane KL type 1; mKL-2, membrane KL type 2. Dotted lines indicate the locations of sequences missing relative to mKL-1. (B) Relative messages of KLs. ECs were infected with lentivirus carrying mKL-1 or mKL-2. Messages of the KLs were quantified by qPCR. The value of the message in ECs was considered as 1.
(TIF)
