# Supplementary figures and images for: c-Kit-Mediated Functional Positioning of Stem Cells to Their Niches Is Essential for Maintenance and Regeneration of Adult Hematopoiesis
Source: PLoS One. 2011 Oct 26;6(10):e26918. doi: 10.1371/journal.pone.0026918 (PMC3202594; doi:10.1371/journal.pone.0026918)

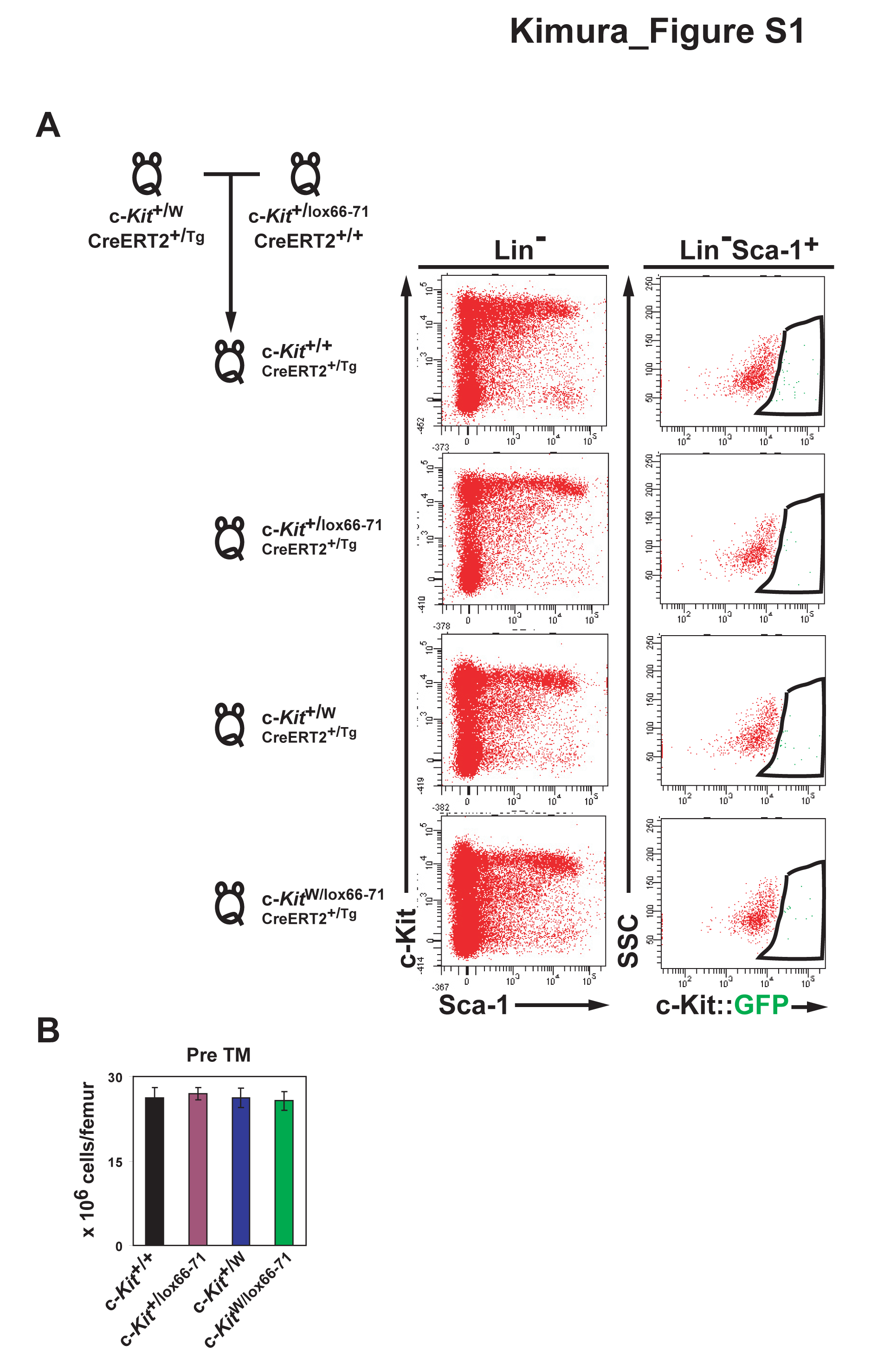

Supplement: Figure S1 — Normal LSK cells in c-Kit conditional KO GFP-reporter mice in pre-activation of Cre recombinase. (A) Normal LSK cells are present in the BM of c-Kit W/lox66-71 mice at the time of pre-treatment with tamoxifen. The schema shows breeding strategy to generate c-Kit W/lox66–71 mice carrying ROSA-CreERT2 transgene as well as control ROSA-CreERT2 c-Kit +/+, ROSA-CreERT2 c-Kit +/lox66–71, and ROSA-CreERT2 c-Kit +/W mice. The flow cytometric analysis demonstrated that normal population of LSK cells manifests no GFP expression in the BM of ROSA-CreERT2 c-Kit +/+, ROSA-CreERT2 c-Kit +/lox66–71, ROSA-CreERT2 c-Kit +/W, and ROSA-CreERT2 c-Kit W/lox66–71 mice. The flow cytometric data are representative of each genotype. (B) Normal number of BMMNCs in femurs of ROSA-CreERT2 c-Kit +/+, ROSA-CreERT2 c-Kit +/lox66–71, ROSA-CreERT2 c-Kit +/W, and ROSA-CreERT2 c-Kit W/lox66–71 mice at the time of pre-treatment with tamoxifen. Data are means ± s.d. (n = 3). (TIF) [file pone.0026918.s001.tif]

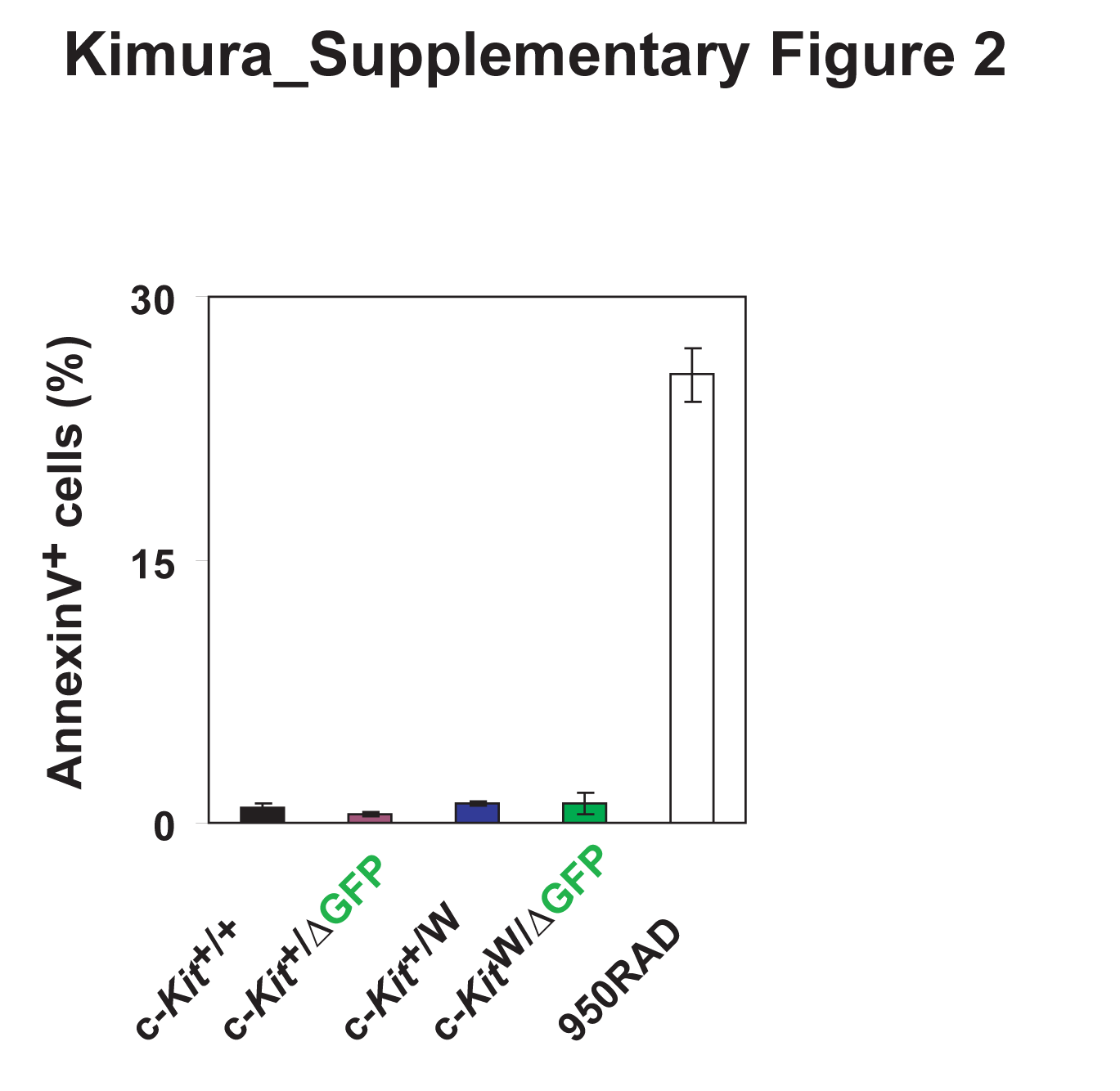

Supplement: Figure S2 — No apoptosis was detected in c-Kit-deficient Lin−Sca-1+c-Kit−c-Kit::GFP+ cells in the BM of c- Kit W/ΔGFP mice. Apoptotic status was examined in LSK or Lin−Sca-1+c-Kit−c-Kit::GFP+ cells in the BM of c-Kit +/+, heterozygous c-Kit +/ΔGFP, heterozygous c-Kit +/W mice, and c-Kit-deficient c-Kit W/ΔGFP mice by staining with AnnexinV. As a positive control, wild-type mice were irradiated with 950 Rads, and BM cells from the mice on day 3 post radiation were analyzed for apoptotic status. Results are mean percentages ± s.d. of AnnexinV+ cells gated on DAPI− cells derived from three mice. (TIF) [file pone.0026918.s002.tif]

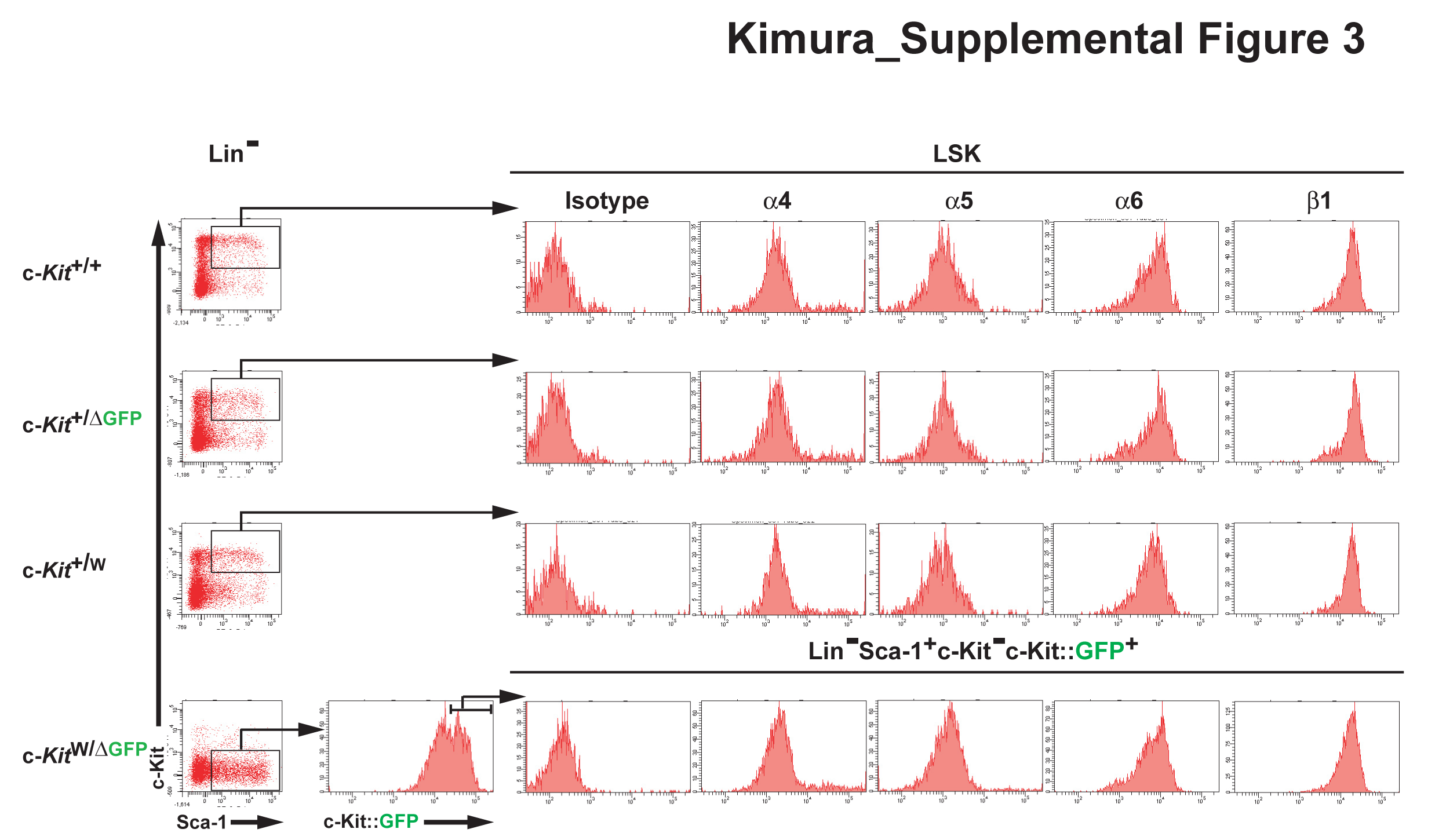

Supplement: Figure S3 — Normal expressions of integrins on HSPCs lacking c-Kit expression. The flow cytometric data show expressions of α4, α5, α6, and β1 integrins on LSK or Lin−Sca-1+c-Kit−c-Kit::GFP+ cells in the BM of c-Kit +/+, heterozygous c-Kit +/ΔGFP, heterozygous c-Kit +/W, and c-Kit-deficient c-Kit W/ΔGFP mice. Data are representative of more than three experiments with three mouse per genotype. (TIF) [file pone.0026918.s003.tif]

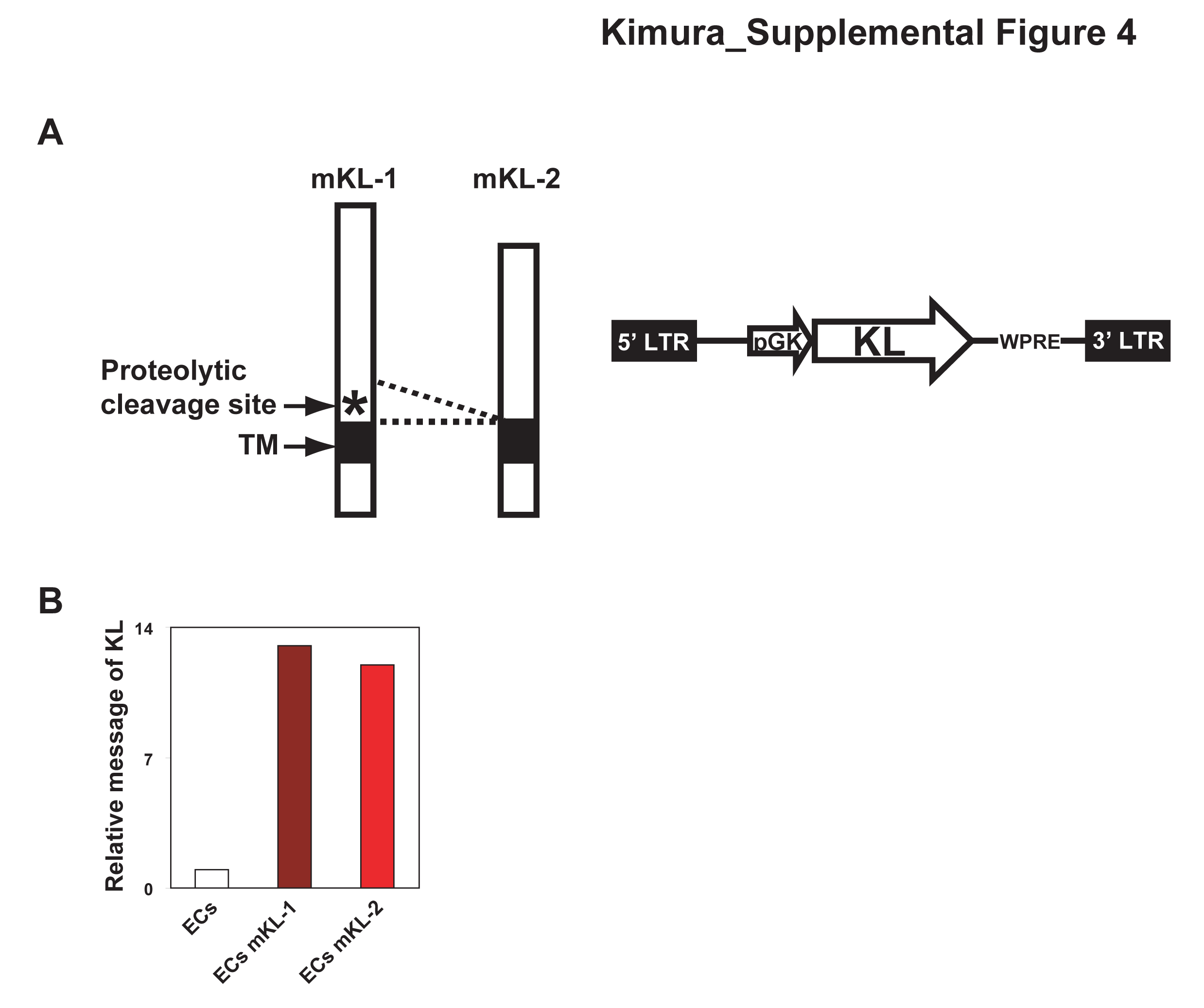

Supplement: Figure S4 — Overexpression of KLs on ECs. (A) Illustrations show diagrammatic structures of series of KLs and a backbone of the lentivirus vectors. An asterisk indicates the position of the proteolytic cleavage site. mKL-1, membrane KL type 1; mKL-2, membrane KL type 2. Dotted lines indicate the locations of sequences missing relative to mKL-1. (B) Relative messages of KLs. ECs were infected with lentivirus carrying mKL-1 or mKL-2. Messages of the KLs were quantified by qPCR. The value of the message in ECs was considered as 1. (TIF) [file pone.0026918.s004.tif]
